# Towards a relational paradigm in sustainability research, practice, and education

**DOI:** 10.1007/s13280-020-01322-y

**Published:** 2020-02-28

**Authors:** Zack Walsh, Jessica Böhme, Christine Wamsler

**Affiliations:** 1 Institute for Advanced Sustainability Studies e.V, Berliner Strasse 130, 14467 Potsdam, Germany; 2 Leuphana University of Lüneburg, Universitätsallee 1, 21335 Lüneburg, Germany; 3 Lund University Centre for Sustainability Studies, Box 170, 221 00 Lund, Sweden

**Keywords:** Complexity, New materialism, Posthumanism, Process philosophy, Relationality, Systems theory

## Abstract

Relational thinking has recently gained increasing prominence across academic disciplines in an attempt to understand complex phenomena in terms of constitutive processes and relations. Interdisciplinary fields of study, such as science and technology studies (STS), the environmental humanities, and the posthumanities, for example, have started to reformulate academic understanding of nature-cultures based on relational thinking. Although the sustainability crisis serves as a contemporary backdrop and in fact calls for such innovative forms of interdisciplinary scholarship, the field of sustainability research has not yet tapped into the rich possibilities offered by relational thinking. Against this background, the purpose of this paper is to identify relational approaches to ontology, epistemology, and ethics which are relevant to sustainability research. More specifically, we analyze how relational approaches have been understood and conceptualized across a broad range of disciplines and contexts relevant to sustainability to identify and harness connections and contributions for future sustainability-related work. Our results highlight common themes and patterns across relational approaches, helping to identify and characterize a relational paradigm within sustainability research. On this basis, we conclude with a call to action for sustainability researchers to co-develop a research agenda for advancing this relational paradigm within sustainability research, practice, and education.

## Introduction

Shifting the paradigms from which systems arise is said to be the most effective leverage point for creating change (Meadows 1999; Abson et al. 2017). Paradigms shape how we perceive the world, what we believe is possible, and how we understand and address sustainability challenges. It is, therefore, critical for sustainability scholars to understand the paradigms shaping their field and to orient their work in line with the most advanced theories and practices from fields relevant to sustainability.

In this paper, we define paradigms as commonly agreed upon ways of perceiving the world based on linked assumptions which have been accepted into the mainstream (Mackinnon and Powell 2008). Mainstream approaches to sustainability currently fall mainly within a technocratic paradigm, focused on addressing certain elements of the system without addressing the intrinsic relations between those elements. System science reveals though, that relations between the elements in the system effect the state of the system as a whole (Kauffman 1995).

Accordingly, various authors have recently argued that a lack of relationality is at the core of many of our current crises, and describe what may be considered an emerging paradigm informed by relational thinking using different terms and concepts, such as the ecological paradigm (Ulanowicz 2009; Hörl 2017), systems approach (Capra and Luisi 2014), integral theory (Wilber 1996), metamodernism (Freinacht 2017), and constructive postmodernism (Cobb 2002). As relationality has become a buzz word with many meanings, however, it is unclear whether different relational thinkers share linked assumptions that constitute an emerging paradigm and to what degree they relate to sustainability.

Against this background, we analyze how relational discourses^1^ have been understood and conceptualized across a broad range of disciplines and contexts relevant to sustainability to identify and harness their connections and contributions for future sustainability-related work. For an emerging paradigm to become mainstream, there must be a coordinated shift in our way of being, thinking, and acting. To better understand how assumptions may be linked, we have, therefore, categorized literature into ways of being (ontologies), thinking (epistemologies) and acting (ethics). These three categories were selected as fundamental aspects of relationality based on the work of Varela (1999), Barad (2007), Kassel et al. (2016), Escobar (2017), and Puis de la Bellacasa (2017) who describe relational ways of being, thinking, and acting as a single tri-partite constellation—an ethico-onto-epistemology—that does not presuppose subject-object and nature-culture binaries.

Accordingly, in this paper, we will identify relational approaches to ontology, epistemology, and ethics which are relevant to sustainability. After describing our method of analysis (“Methodology”), we present what relational approaches to ontology encompass (“Relational Approaches to Ontology”), how relational approaches to epistemology can shape research practice (“Relational Approaches to Epistemology”), and the normative, ethical orientations underlying relational approaches to sustainability (“Relational Approaches to Ethics”). On this basis, we discuss the identified trends, themes, and patterns characterizing a relational approach to sustainability, concluding with recommendations for future research (“Conclusions”).

## Methodology

This study presents a qualitative literature review to analyze how relational approaches relevant to sustainability have been understood and conceptualized. Indications of a relational paradigm come from diverse systems of knowledge in the humanities, social sciences, and natural sciences. Academic literature across multiple disciplines was selected for analysis insofar as they discussed relational approaches to ontology, epistemology, and ethics and were related to the context of sustainability.

Literature was selected based on an exploratory approach, combining the use of scholarly database searches (e.g. Scopus and Google Scholar) with a consultation process with different key stakeholders and informants.^2^ The latter involved a total of five workshops and continuous communication with participants through the participatory development of a web-based communication platform and database in the field between 2017 and 2019.^3^ This resulted in the identification of a total of 100 publications for analysis (cf. “Relational Approaches to Ontology”, “Relational Approaches to Epistemology” and “Relational Approaches to Ethics”). The categorization of the identified papers to the three categories (ontology, epistemology and ethics) was based on the following definition of these terms and their relevance for sustainability:

(A) *Ontologies* describe the “assumptions (which may be implicit or explicit) about what kinds of things do or can exist in [reality], and what might be their conditions of existence, relations of dependency, and so on” (Scott and Marshall 2009, p. 531).
(B) *Epistemologies* describe how we come to know the world. They define the criteria, standards, and methods for understanding reality (Steup 2018).
(C) *Ethics* describes “what is morally good and bad and morally right and wrong” (Singer 2019, para. 1). It includes cultural values, morals, and norms shaped by social and political life.

**Fig. 1:**
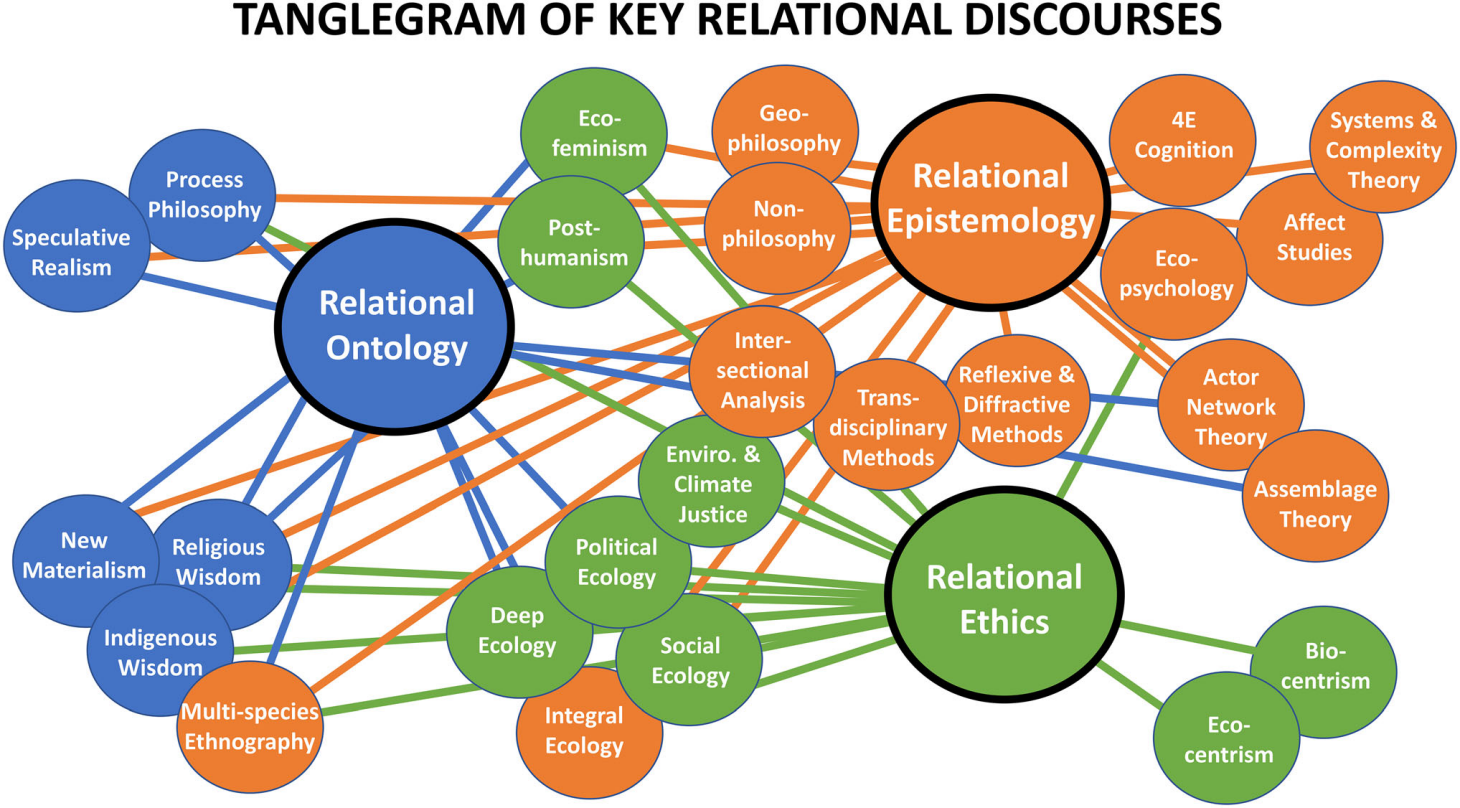
Functional assemblage of twenty-six relational discourses relevant to sustainability with connections to ontology, epistemology, and ethics

These 3 categories were separated for the purposes of presenting a clear analysis, while acknowledging that the categories and discourses are mutually entangled. As such, the categorization schema is a fuzzy set^4^ which assigns discourses membership to a primary category while acknowledging that they relate to more than their assigned category.^5^ We separate discourses to highlight specific relationships that could prove helpful in further developing relational approaches to sustainability, whilst we recognize that discourses could be differently categorized, allowing new relationships to become visible. What we construct is therefore one potential functional assemblage that may be explored in future sustainability research. Figure 1 presents a tanglegram (Hodder 2012), highlighting the identified entanglements of the 26 most prominent discourses outlined in our analysis (“Relational Approaches to Ontology”, “Relational Approaches to Epistemology” and “Relational Approaches to Ethics”).^6^ The tri-partite categorization offers a functional framework for developing relational approaches to sustainability in concert with each other, drawing upon the diversity of discourses while respecting both their distinctions and intra-relations.

## Relational approaches to ontology

A total of 25 publications were identified as relevant regarding relational approaches to ontology. They come mainly from the fields of philosophy, indigenous and religious studies, cultural studies, and political science. In this context, relevant discourses describing relational ontologies relate to speculative realism, process philosophy, new materialism, indigenous wisdom, and religious wisdom (Fig. 1). All relational ontologies posit that “the relations between entities are more fundamental than the entities themselves” (Wildman 2006, p. 1). No entity preexists the relations that constitute it.

Within the identified literature, the majority of sources describe relational ontologies that can be broadly categorized as either undifferentiated or differentiated. Undifferentiated relational ontologies are monistic, viewing an entity as “an evolving expression of a metaphysical source” (Stout 2012, p. 389). Ecological holism is a form of undifferentiated relational ontology, for example, that dissolves the distinctions between mind, matter, and life in terms of more fundamental activities of the universe (Smuts 1926). By contrast, differentiated relational ontologies view reality as an evolving unique expression of complex, relational, multidimensional sources (Stout 2012, p. 389). The latter conceives identity and difference in relation to each other, whereas the former assimilates differences in more fully encompassing forms of identity. The difference between undifferentiated and differentiated relational ontology is consequential for sustainability research. White et al.’s (2016) comprehensive survey of hybrid theoretical approaches to society and nature demonstrates the importance of taking a differentiated relational approach, so as to understand the mutual relations between social and ecological systems without dichotomizing or subsuming one into the other.

Contemporary discourses on relational ontology in Western thought were identified as belonging to speculative realism, process philosophy, and new materialism. Speculative realism (hereafter SR) is a heterogenous body of thought in which various philosophies posit very different alternatives to the bifurcation of nature/culture and the anti-realism of modern Enlightenment philosophy. SR’s core commitments are to a renewed willingness to entertain speculative metaphysics and ontological realism in an attempt to overcome the problem of *correlationism*. As most famously described by Kant, correlationism posits that an object cannot be known outside its relationship to the mind, such that knowledge of reality is always a correlation between thinking and being (Bryant et al. 2011). SR seeks various ways to describe reality outside this contradiction.

Process philosophy is an antecedent of SR known to possess a differentiated relational ontology (Keller and Daniell 2002; Faber and Stephenson 2011; Shaviro 2014). The progenitor of process philosophy, Alfred North Whitehead (1929), posited that every actual entity composes societies of ever-greater societies, while being both internally related and differentiated from other actual entities. The social, he claimed, “is a way of describing how each entity is constituted by and through its environment” (Halewood 2011, p. 121). Recent works by Henning (2005), Ims et al. (2015), Stengers (2015), Muraca (2016), Latour (2017), Kaaronen (2018), and Mancilla et al. (2019) demonstrate the multiple ways process-relational ontologies shift epistemological and ethical orientations to human–nature interactions based on an understanding of their co-constitution. Latour (2017) is probably one of the best-known authors writing about process philosophy and ecology who argues that the Earth should be conceived as a complex assemblage of living and agential processes which should be given political standing.

Another heterogenous body of thought that develops relational approaches to ontology in the context of sustainability is new materialism. New materialism makes a core commitment to experiment with post-Cartesian ontologies that explore the variegated relationships between different nature-cultures. New materialists generally employ multi-modal methodologies that examine various levels (micro-, meso-, and macro-) of socio-ecological systems simultaneously (Coole and Frost 2010). Jane Bennet is, for instance, one of the better-known new materialists. In *Vibrant Matter* (2010), she develops a “vibrant materialism” that (like Latour) attributes agency to nonhumans, and that (like Whitehead) views living and non-living matter as co-constituting assemblages.

These discourses on relational ontology (SR, process philosophy, and new materialism) are comparatively recent developments emerging within Western thought. Most relational ontologies have, however, developed historically outside the West for millennia (Todd 2016). Worldwide, there are many non-modern, earth-based, indigenous and religious ontologies that never inherited the bifurcation of nature/culture characteristic of the Western modern worldview. These traditions all focus on the inter-related, inter-dependent, and inter-active aspects of nature-cultures. Unlike Western environmentalism, these traditions do not relate to the environment as something ‘out there’ that needs to be protected. Landscapes are considered both physical and mental phenomena, bearing the markings of personal and collective biographies, task-scapes, customs, rituals, and cosmologies (Miller et al. 2014; Miller 2017). Indigenous peoples of the Americas, for example, follow a relational ontology based on kinship. They perceive themselves and nature as part of the same family sharing origins and ancestral bonds (Salmon 2000; Datta 2015; Posthumus 2018).

## Relational approaches to epistemology

A total of 52 publications were identified as relevant regarding relational approaches to epistemology. They come mainly from the fields of cognitive science, psychology, sociology, philosophy, science and technology studies, feminism, and sustainability science. Relevant discourses describing relational epistemologies within the identified literature relate to 4E cognition, affect studies, ecopsychology, assemblage theory, actor-network theory (ANT), multi-species ethnography, integral ecology, geo-philosophy, non-philosophy, transdisciplinary (TD) methods, intersectional analysis, systems and complexity theory, and reflexive and diffractive methods (Fig. 1).

There is broad consensus that modern western epistemologies arising from the Enlightenment and scientific revolution are largely responsible for creating profound divisions and patterns of exploitation between humans and nonhumans. Their intellectual foundations were formed by figures such as Isaac Newton, Immanuel Kant, David Hume, John Locke, Francis Bacon, and René Descartes (Griffin 2001). They posit: (1) The idea that causation is determined only by external relations between objects; (2) that no object can be understood outside its relation to thought; (3) that primary and secondary (sensible) qualities are separable and that science can objectively study the former without the latter; (4) that nature can be mastered, ‘her’ secrets revealed to instrumental reason and scientific ‘progress’; and finally, (5) that mind and body are separable substances, and that the latter is the domain of objective scientific inquiry. These ideas formed the philosophy of empiricism that shaped the development of science, technology, and industry throughout the modern period. Though these ideas have been profoundly influential in shaping society, as Latour (1991) argues, we have never been truly modern. Despite modern people believing nature could be understood objectively, scientific knowledge is fundamentally shaped by social relations and practices. Researchers have always shaped and been shaped by the objects of their research. As such, many researchers now increasingly use reflexive methods to account for the observer’s role in shaping knowledge (May and Perry 2017).

In this context, the identified relevant literature from the field of cognitive science uses embodied, embedded, extended, and enactive (4E) approaches to cognition to scientifically understand the complex and dynamic interactions between coupled brain–body–environment systems (Varela et al. 1991; Clark 2008). Evan Thompson (2010), for instance, argues that closing the explanatory gap between consciousness and life is possible by incorporating phenomenological accounts of experience into scientific accounts of mind and life. Frequently, 4E approaches are also called 4EA, so as to include the growing field of affect studies (Gregg and Seigworth 2010)—an interdisciplinary body of research taking relational approaches to emotions (Slaby 2016) that has examined emotional relationships to environments (Bladow and Ladino 2018), media ecology (Angerer 2017), and body politics (Protevi 2009).

The review of relevant literature in psychology stipulates that identity-based, value-based, and socio-cognitive approaches provide the best ways of bridging knowledge of personal and social-ecological transformation (Bögel and Upham 2018, p. 18). Ecopsychology is a branch of psychology that draws upon the ecological sciences to study the constitutive relations between minds and environments (Kanner et al. 1995; Fisher 2013). Studies on ecopsychology are typically concerned with the ecological unconscious, phenomenology, the interconnectedness of all beings, the transpersonal, and the transcendental (Kahn and Hasbach 2012).

The review of the identified social scientific literature shows a growing interest in relational approaches to knowing. These approaches allow social scientists new methods for analyzing human-nonhuman relations. Assemblage theory (DeLanda 2006) considers all things living and non-living to be assemblages of human and nonhuman parts. Several methods for studying assemblages have developed in empirical work (e.g. McFarlane 2011; Baker and McGuirk 2017; Feely 2019). Actor-network theory (ANT) is among the relational methods most frequently used in the social sciences (Latour 2005). It does not position humans at the center or apex of agency and responsibility, but rather, considers agency to be distributed among various actants—none of which are themselves solely responsible for change. It studies how agency is formed by an interlinked chain of beings and processes, rather than any individual. To write about agency outside humanist epistemology, scholars frequently employ multi-species ethnography (e.g. Kirksey and Helmreich 2010; Kirksey 2014; Multispecies Editing Collective 2017).

In the field of philosophy, our review shows that relational epistemologies are being developed to help us think transversally across different geo-social scales. Integral approaches to ecology, also known as integral ecology, cross-boundaries between the humanities, social sciences, and natural sciences (e.g. Esbjörn-Hargens and Zimmerman 2009; Mickey 2014; Mickey et al. 2017). O’Brien and Hochachka (2010), for example, use integral theory to develop a multi-disciplinary, multi-perspectival understanding of climate change adaptation. Deleuze and Guattari’s geo-philosophy is another approach to traversing mental, social and environmental ecologies (Bonta and Protevi 2004), as is Francois Laruelle’s non-philosophy, which provides a method for different ways of knowing (e.g. theologically, philosophically, and scientifically) to inform each other without imposing hierarchies (Smith 2013). These emerging philosophical approaches offer ways to think ecologically; not just to think ‘about ecology,’ but rather to think in terms of a ‘general ecology’ (Hörl 2017). Morton (2013, 2016) exemplifies work in this mode. He defines ecological awareness as a knowing that loops in on itself, as in a meditation, where one becomes familiar with ‘the mesh’ of inter-related happenings and their constitutive relations to oneself.

Transdisciplinary sciences have also begun developing relational approaches to knowing (Nicolescu 2002; Craps and Brugnach 2015; Van Breda and Swilling 2018). Systems theory (incl. general systems theory, cybernetics, and complexity theory) is among the most prevalent discourses within these sciences (cf. Barile et al. 2018; Preiser et al. 2018). According to Capra and Luisi (2014), systems thinking developed in the 1920s by biologists, Gestalt psychologists, ecologists, and quantum physicists. It is characterized by several important shifts of perspective: from the parts to the whole; from disciplines to multidisciplinarity; from objects to relationships; from measuring to mapping; from quantities to qualities; from structures to processes; from objective to epistemic science; and from Cartesian certainty to approximate knowledge (pp. 80–82).

Feminist scholars offer important socially situated epistemological discourses, including standpoint theory (Harding 1991), situated knowledge (Haraway 1988), and intersectional analysis (Crenshaw 1989). These discourses politicize and ethically orient sustainability research and have been most frequently employed within environmental justice scholarship (e.g. Kaijser and Kronsell 2014; Malin and Ryder 2018). Feminist scholars have also developed diffractive methods to overcome the shortcomings of reflexive methods (e.g., Barad 2007; Bozalek and Zembylas 2017; Hill 2017). Diffractive methods are used to read the insights of one discipline through another discipline to generate novel insights in the relation between differences (e.g., Larson and Philips 2013; Massei 2014; Doucet 2018; Gullion 2018).

Finally, our review shows that in the field of sustainability science, scholars increasingly call for developing empirical methods that account for subjectivity and its role in shaping scientific practice (cf. Wamsler et al. 2018). Manuel-Navarrete (2015) claims for instance that research on ‘mind maps’ and ‘mental models’ provide generalizable ways of objectively analyzing subjectivity and integrating it in systems research and institutional arrangements.

## Relational approaches to ethics

A total of 23 publications were identified as relevant regarding relational approaches to ethics. They come mainly from the fields of sustainability science, philosophy, religious studies, and cultural studies. Relevant discourses describing relational approaches to ethics within the literature studied include biocentrism, ecocentrism, deep ecology, social ecology, political ecology, environmental and climate justice, ecofeminism, and posthumanism (Fig. 1). The latter five discourses have been provisionally included under the category of ethics. Although they have shaped understandings of ontology and epistemology, they are nevertheless normative discourses influencing values, morals, and norms, especially at a societal level.

The identified dominant relational approaches to ethics within the fields of environmental and climate ethics include biocentrism and ecocentrism. Biocentrism and ecocentrism attribute moral significance to biological organisms and ecological systems, respectively. Collectively, they are committed to non-anthropocentrism, meaning that they do not position human interests at the center of moral concern.^7^

Deep ecology is an influential discourse, emphasizing the need to shift consciousness as a prerequisite for shifting modern industrial society toward a more sustainable paradigm. It was coined by the Norwegian eco-philosopher Arne Naess. Naess contrasts deep ecology with shallow ecology, arguing that whereas the latter views nature anthropocentrically in terms of nature’s utility for us, deep ecology mines resources from spiritual, religious, and philosophical traditions to view nature eco-centrically. Although there can be many different versions of deep ecology, Naess’ version (ecosophy ‘T’) is informed by Spinoza, Mahayana Buddhism, and the Gandhian philosophy of non-violence. As conflicts of interest arise, the health and flourishing of humans and nonhumans are considered holistically, such that the vitality of higher-order (more complex) systems is protected over that of lower-order systems (Drengson and Devall 2010).

Critical scholars contend that deep ecology has an apolitical view of systems change, so they claim it is important to integrate deep ecology with social ecology (Slocombe 2002). Gary Snyder is one example of a thinker who has integrated both deep and social ecology in his activism and writings (Messersmith-Glavin 2012). As developed by Bookchin (Biehl 1999), social ecology adds a critical perspective on class-based struggles of marginalized people by considering how ecology is informed by social hierarchy and domination. Radical social ecology investigates the material, social, and spiritual conditions of an ecological society by pursuing the elimination of human’s domination of nature via the elimination of human’s domination of humans. It connects ecological issues to a broad array of interconnected social issues (Bookchin 1980).

Similarly, political ecology examines asymmetrical distribution of resources and power, helping to address the structural causes, not symptoms of sustainability challenges (Robbins 2012). Environmental and climate justice scholarship applies the methods of intersectional analysis in social and political ecology to the modern environmental movement. By forming alliances with marginalized groups, environmental and climate justice activists and scholars integrate personal and socio-ecological transformation by addressing both social justice issues (especially race, gender, and class-based injustice) in relation to ecological issues (such as air pollution, waste disposal, and access to clean water) (Carder n.d; Mohai et al. 2009).

Among the identified literature from social and political ecology, ecofeminism is among the most important and influential discourses. Ecofeminism “seeks to understand the interconnected roots of all domination,” connecting the oppression and domination of women in particular and marginalized groups in general to the oppression and domination of nature (Plant n.d., p. 101). Plumwood (1993) connects the logic of domination to dualistic structures of reasoning in Western thought. Male/female, mind/body, civilized/primitive, and human/nature dualisms, she argues, naturalize unequal and exploitative relationships based on the domination of subordinate groups. Other noted ecofeminists like Merchant (1980) and Shiva (1989) document how science, technology, and economic development espouse ideas of progress tied to the control and mastery of nature and of women; while spiritually informed ecofeminists such as Ruether (1992, 2005) develop religious responses to these critiques, emphasizing the liberative potential of cultivating feminine principles in society.

In making the claim that women are closer to nature, however, some (but by no means most) ecofeminists have problematically upheld gendered concepts of nature that fail to overcome the dualistic thinking underlying the logic of domination (Gaard 2011). Ecofeminism has since become more critical, intersectional, materialist, and posthumanist (Alaimo and Hekman 2008; Gaard 2017). Prominent recent works include Alaimo (2010), Braidotti (2013), Zylinska (2014), Haraway (2016), Keller (2017) and Puis de la Bellacasa (2017). Posthuman feminists reject essentialist concepts of gender, and are much more technomaterialist, viewing human–nonhuman relations as materially informed by socio-technical systems. Posthumanism does not relegate its interest to animal (zoologic) encounters but explores relations of all kinds—both between biological beings (such as symbionts or holobionts) and cyborgs (or flesh machines).

## Discussion and Conclusions

Our review of the existing bodies of literature that take relational approaches to ontology, epistemology, and ethics relevant for sustainability has identified important developments, common themes, and patterns that constitute characteristics of a relational paradigm (and possible shift towards a relational paradigm) in sustainability research. Despite differences between the various perspectives cited, all describe a paradigm that (i) is grounded in a relational ontology, (ii) emphasizes the need for understanding human and non-human nature as mutually constitutive, and (iii) values more-than-human relations.

Our analysis shows that *relational ontologies* aim to overcome the bifurcation of nature/culture and various other dualisms (e.g. mind/matter, subjectivity/objectivity) shaping the modern worldview. Differentiated (as opposed to undifferentiated) relational ontologies respect the integrity of individuals while understanding how their being is fundamentally constituted by relations of all kinds. In this context, speculative realism, process philosophy, new materialism, and indigenous and religious wisdom traditions are systems of knowledge providing particularly well-developed understandings of relational ontology relevant to sustainability.

Our review also shows that *relational approaches to epistemology* account for the observer’s role in shaping knowledge; acknowledge that agency is distributed across networks; view objects as assemblages of humans and nonhumans; increasingly focus on transdisciplinary methods to cut across disciplinary boundaries; and use diffractive methods to integrate different ways of knowing.

Lastly, our review shows that *relational approaches to ethics* include non-anthropocentric perspectives; value non-human nature in non-instrumental terms; use intersectional methods to analyze the inter-relations between social and ecological issues; and contextualize human–nature interactions in light of asymmetrical power relations and dynamics between assemblages or networks of interest.

This paper discretely analyzed relational approaches to ontology, epistemology, and ethics in an attempt to outline avenues to further develop them as a tri-partite constellation in future sustainability research, practice, and education.^8^ Accordingly, the results and the developed analytical tri-partite framework on which they were based, can enable scholars and practitioners to identify and harness the contributions of relational approaches to sustainability in a more systematic way.

Currently, there exist only a few studies that explicitly take, to some extent, relational approaches to sustainability. These include research in fields, such as resilience (e.g. Darnhofer et al. 2016; Lejano 2019); socio-technical transitions (e.g. Garud and Gehman 2012; Chilvers and Longhurst 2015; Haxeltine et al. 2017); sustainability education (e.g. Netherwood et al. 2006; Williams 2013; Lange 2018; O’Neil 2018; Mcphie and Clarke 2019; Taylor and Pacini-Ketchabaw 2019); environmental values (e.g. Jax et al. 2018; Pascual et al. 2018; Saxena et al. 2018); posthuman sustainability (e.g. Cielemęcka and Daigle 2019; Fox and Alldred 2019; Smith 2019); and quantum theory in sustainability (e.g. O’Brien 2016; Rigolot 2019). In spite of such exceptions, few sustainability researchers make explicit the related discourses outlined in this paper.

In fact, our analysis shows that relational approaches are marginalized within sustainability scholarship, despite the broad academic interest in relationality emerging across other fields. This article, therefore, calls scholars to consider the identified discourses in future sustainability research, practice, and education.

The identified relational approaches provide a basis for integrating so-called “inner” and “outer,” “personal” and “collective” dimensions of sustainability without presupposing the logic of dualism underlying that language and framing. Ives et al. (2019) recently called for exploring relations among these dimensions, rather than discussing them as discrete dimensions.

Based on our results, we call for further research to better understand the generative interconnections between these various discourses and dimensions. More specifically, we call for further research that investigates how relational ontologies, epistemologies, and ethics intra-act to compose a relational approach to sustainability. In this context, intra-action means “*the mutual constitution of entangled agencies*. That is, in contrast to the usual ‘interaction,’ which assumes that there are separate individual agencies that precede their interaction, the notion of intra-action recognizes that distinct agencies do not precede, but rather emerge through, their intra-action” (Barad 2007, p. 33). On this basis, we conclude with a call to action for sustainability scholars and practitioners to co-develop a research agenda for advancing a relational paradigm within sustainability research, practice, and education based on relational ways of being, knowing, and acting.

## Footnotes

1 The term ‘discourse’ defines ways to think and communicate about a given subject matter.

2 The key stakeholders were identified though a targeted selection of scholars and practitioners and an open call for participation related to the themes of this paper. The workshops included a total of 125 participants.

3 http://www.ama-project.org/.

4 Zadeh (1965) defines fuzzy sets as “a class of objects with a continuum of grades of membership. Such a set is characterized by a membership (characteristic) function which assigns to each object a grade of membership ranging between zero and one” (p. 338).

5 For instance, posthumanism and ecofeminism make ontological and epistemological critiques, not just ethical ones; nevertheless, they have been included under ethics because unlike other discourses, they are explicitly normative in orientation.

6 Although certain discourses have been clustered together to designate their relative affinity, the distance between discourses and the 3 categories is insignificant.

7 Non-anthropocentric approaches to environmental and climate ethics are collected in Henning and Walsh (2020).

8 The web-based platform, upon which this research is partly based, has been developed to support such a task: http://www.ama-project.org/.
